# Effect of Sleep Extension on Objectively Assessed Energy Intake Among Adults With Overweight in Real-life Settings: A Randomized Clinical Trial

**DOI:** 10.1001/jamainternmed.2021.8098

**Published:** 2022-02-07

**Authors:** Esra Tasali, Kristen Wroblewski, Eva Kahn, Jennifer Kilkus, Dale A. Schoeller

**Affiliations:** 1 Department of Medicine, The University of Chicago, Chicago, Illinois; 2 Department of Public Health Sciences, The University of Chicago, Chicago, Illinois; 3 Biotechnology Center, Department of Nutritional Sciences, University of Wisconsin–Madison, Madison

## Abstract

**Importance:**

Short sleep duration has been recognized as a risk factor for obesity. Whether extending sleep duration may mitigate this risk remains unknown.

**Objective:**

To determine the effects of a sleep extension intervention on objectively assessed energy intake, energy expenditure, and body weight in real-life settings among adults with overweight who habitually curtailed their sleep duration.

**Design, Setting, and Participants:**

This single-center, randomized clinical trial was conducted from November 1, 2014, to October 30, 2020. Participants were adults aged 21 to 40 years with a body mass index (calculated as weight in kilograms divided by height in meters squared) between 25.0 and 29.9 and had habitual sleep duration of less than 6.5 hours per night. Data were analyzed according to the intention-to-treat principle.

**Interventions:**

After a 2-week habitual sleep period at baseline, participants were randomized to either an individualized sleep hygiene counseling session that was intended to extend their bedtime to 8.5 hours (sleep extension group) or to continue their habitual sleep (control group). All participants were instructed to continue daily routine activities at home without any prescribed diet or physical activity.

**Main Outcomes and Measures:**

The primary outcome was change in energy intake from baseline, which was objectively assessed as the sum of total energy expenditure and change in body energy stores. Total energy expenditure was measured by the doubly labeled water method. Change in body energy stores was computed using regression of daily home weights and body composition changes from dual-energy x-ray absorptiometry. Sleep duration was monitored by actigraphy. Changes from baseline were compared between the 2 groups using intention-to-treat analysis.

**Results:**

Data from 80 randomized participants (mean [SD] age, 29.8 [5.1] years; 41 men [51.3%]) were analyzed. Sleep duration was increased by approximately 1.2 hours per night (95% CI, 1.0 to 1.4 hours; *P* < .001) in the sleep extension group vs the control group. The sleep extension group had a significant decrease in energy intake compared with the control group (−270 kcal/d; 95% CI, −393 to −147 kcal/d; *P* < .001). The change in sleep duration was inversely correlated with the change in energy intake (*r* = −0.41; 95% CI, −0.59 to −0.20; *P* < .001). No significant treatment effect in total energy expenditure was found, resulting in weight reduction in the sleep extension group vs the control group.

**Conclusions and Relevance:**

This trial found that sleep extension reduced energy intake and resulted in a negative energy balance in real-life settings among adults with overweight who habitually curtailed their sleep duration. Improving and maintaining healthy sleep duration over longer periods could be part of obesity prevention and weight loss programs.

**Trial Registration:**

ClinicalTrials.gov Identifier: NCT02253368

## Key Points

**Question:**

What is the effect of sleep extension on objectively assessed energy intake in adults with overweight in their usual home environment?

**Findings:**

In this randomized clinical trial of 80 adults with overweight and habitual sleep less than 6.5 hours per night, those randomized to a 2-week sleep extension intervention significantly reduced their daily energy intake by approximately 270 kcal compared with the control group. Total energy expenditure did not significantly differ between the sleep extension and control groups, resulting in a negative energy balance with sleep extension.

**Meaning:**

The findings suggest that improving and maintaining adequate sleep duration could reduce weight and be a viable intervention for obesity prevention and weight loss programs.

## Teaser

This randomized clinical trial examines the energy intake, energy expenditure, and body weight in adults who slept less than 6.5 hours per night.

## Introduction

Obesity is a major public health concern.^1^ The obesity epidemic appears to coincide with a pattern of sleeping less that has been observed in society over the past several decades. For example, one-third of the US population reported not getting the recommended 7 to 9 hours of sleep per night.^2,3,4^ Substantial evidence suggests that sleeping less than 7 hours per night on a regular basis is associated with adverse health consequences.^5^ Particularly, insufficient sleep duration has been increasingly recognized as an important risk factor for obesity.^6,7^ Prospective epidemiologic studies suggest that short sleep duration is an important risk factor for weight gain.^8,9,10^ However, it remains unknown whether extending sleep duration can be an effective strategy for preventing or reversing obesity. Although sleep hygiene education is encouraged by obesity experts,^11^ most health professionals and patients do not implement obtaining adequate sleep duration as part of the strategies to combat the obesity epidemic.^12^

At the population level, the association between energy flux and body weight implicates that increased energy intake is the main factor in higher body weights in modern society.^13^ According to dynamic prediction models, a sustained increase in energy intake of even 100 kcal/d would result in a weight gain of about 4.5 kg over 3 years.^14,15^ Factors that underlie the observed persistent increase in energy intake and mean weight gain at the population level need to be better understood. One such factor is insufficient sleep duration. Short-term experimental laboratory studies have found that sleep restriction in healthy individuals is associated with an increased mean energy intake of about 250 to 350 kcal/d with minimal to no change in energy expenditure.^16,17,18,19^ However, these laboratory studies do not represent real life. The magnitude of sleep restriction was extreme in most cases, and energy intake was ascertained from a single or a few meals. In a real-life setting in which participants continue their normal daily activities, multiple interacting factors (eg, social interactions and free-living physical activity) can influence energy intake or expenditure and weight.

To date, it remains unknown whether and to what extent an intervention that is intended to increase sleep duration in a real-life setting affects energy balance and body weight. We conducted a randomized clinical trial (RCT) to determine the effects of a sleep extension intervention on objectively assessed energy intake, energy expenditure, and body weight in real-life settings among adults with overweight who habitually curtailed their sleep duration.

## Methods

### Study Design, Setting, and Participants

This single-center, parallel-group RCT was conducted from November 1, 2014, to October 30, 2020. The protocol was approved by The University of Chicago Institutional Review Board, and participants provided written informed consent. The study protocol is available in Supplement 1. We followed the Consolidated Standards of Reporting Trials (CONSORT) reporting guideline.

Adult men and women aged 21 to 40 years with a body mass index (calculated as weight in kilograms divided by height in meters squared) between 25.0 and 29.9 and a mean habitual sleep duration of less than 6.5 hours per night were eligible. Individuals were required to have stable self-reported sleep habits for the past 6 months. They were recruited from the community and completed an initial online survey followed by a face-to-face interview. Race and ethnicity data were self-reported at this time and included the following race and ethnicity categories: Asian, Black or African American, Hispanic, and White. Those who met the inclusion criteria underwent laboratory screening (polysomnography, oral glucose tolerance test, and blood tests) to determine eligibility. Habitual sleep duration was confirmed by a 1-week screening wrist actigraphy at home. Those who had obstructive sleep apnea confirmed by laboratory polysomnography (apnea-hypopnea index >5), insomnia or history of any other sleep disorder, or night shift and rotating shift work (current or in the past 2 years) were excluded. Detailed eligibility criteria are provided in the eMethods in Supplement 2.

### Blinding and Randomization

**Figure 1.**
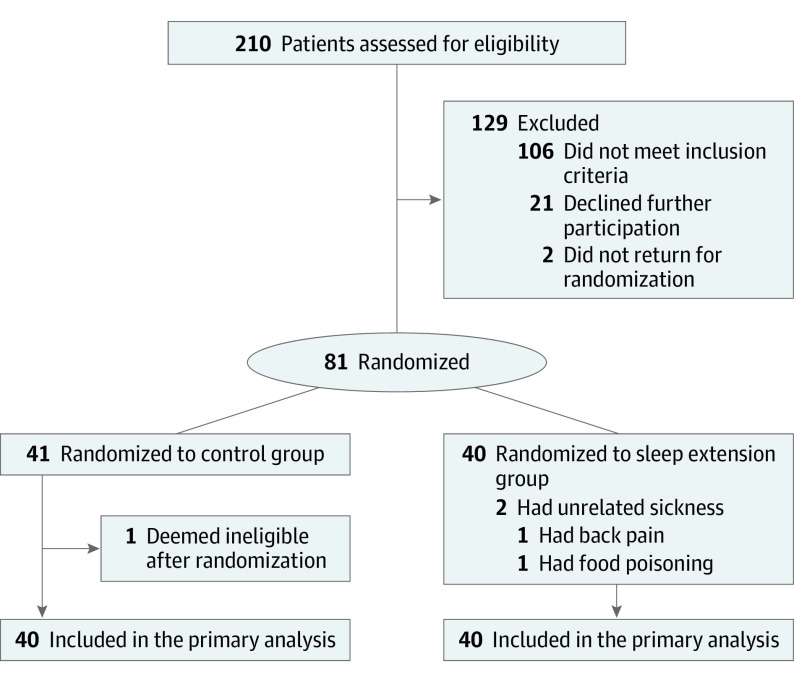
CONSORT Flow Diagram

After a 2-week habitual sleep period at baseline, participants were randomized to either 2-week sleep extension (sleep extension group) or 2-week continued habitual sleep (control group) (Figure 1). Participants continued their daily routine activities at home without any prescribed diet or physical activity.

To blind participants to the sleep extension intervention, we described the study in the recruitment materials as follows: “we will collect information about sleep habits and metabolism.” The sleep extension group was blinded to randomization until after the 2-week baseline assessments, and the control group was blinded until the end of the 4-week study. This approach allowed us to capture habitual sleep-wake patterns without influencing participants' usual behavior or creating selection bias with only participants interested in improving sleep habits. After study completion, all participants were provided with information about the health benefits of optimal sleep duration. Block randomization, stratified by sex, was performed using computer-generated random numbers. Before the trial, randomization assignments were prepared by a biostatistician (K.W.) using opaque, sealed, and numbered envelopes and were given to the research coordinator (E.K.).

### Sleep Monitoring and Intervention

Sleep-wake patterns were continuously monitored at home by wrist actigraphy throughout the 4-week study. Participants were asked to wear an accelerometer (motion)-based monitor (Actiwatch Spectrum Plus; Philips) and to press a built-in event marker button when they went to bed to sleep each night and when they got out of bed each morning. Sleep was automatically scored (Actiware, version 6.0.9; Philips) using validated algorithms as the sum of all epochs that were scored as sleep during the total time spent in bed.^20,21^

During the 2-week baseline, all participants were instructed to continue their habitual sleep patterns at home. On the morning of day 15, participants met with study investigators (E.T. and E.K.) in the research center. Those who were randomized to the sleep extension group received individualized sleep hygiene counseling through a structured interview (E.T.) (eMethods in Supplement 2).^22^ At the end of the interview, participants were provided with individualized recommendations to follow at home for 2 weeks, with the aim of extending their bedtime duration to 8.5 hours. On day 22, participants returned for a brief follow-up visit. Actigraphy data from the first intervention week were reviewed, and further sleep counseling was provided as needed.

To minimize any imbalance in contact with the investigators between the 2 groups, we asked participants in the control group to meet with the study investigators on days 15 and 22. Actigraphy data of these participants were downloaded, but the participants did not receive any specific sleep recommendations and were instructed to continue their daily routine and habitual sleep behaviors until the end of the study.

### Assessments of Energy Intake, Energy Expenditure, and Body Weight and Composition

For each 2-week period, the energy intake was calculated from the sum of total energy expenditure and change in body energy stores using the principle of energy balance.^14,23,24^ Total energy expenditure was measured by the doubly labeled water method.^25,26,27,28,29^ For each 2-week period, the change in body energy stores was computed from the regression (slope, grams per day) of daily home weights and change in body composition (ie, fat mass and fat-free mass) using dual-energy x-ray absorptiometry. Participants were provided a cellular-enabled weight scale (BodyTrace; BodyTrace Inc) and instructed to take their nude weights twice every morning after awakening before eating or drinking. Weight values were hidden from the participants to minimize potential influence on behavior. Changes in body composition were converted to changes in energy stores using 9.5 kcal/g as the energy coefficient of fat mass and 1.0 kcal/g as the energy coefficient of fat-free mass.^30^ Resting metabolic rate was measured by indirect calorimetry for 30 minutes after fasting and for 4 hours after eating a standardized breakfast. Thermic effect of the meal was calculated, which was previously described elsewhere.^31^ Activity energy expenditure was calculated by subtracting the resting metabolic rate and thermic effect of the meal from the total energy expenditure.^31,32^ Additional details are provided in the eMethods in Supplement 2.

### Statistical Analysis

The primary outcome was change in energy intake from baseline. A total final sample size of 80 participants (40 per group) was originally planned and provided 80% power to detect a true difference in energy intake between groups of 207 kcal/d using a 2-sided α = .05 significance threshold (trial protocol in Supplement 1). An intention-to-treat analysis was conducted in Stata, version 16 (StataCorp LLC) using 2-tailed tests with statistical significance set at *P* < .05. Categorical data are presented as counts and percentages. Continuous data are presented as means and SDs. Linear mixed-effects models were fit to determine the treatment differences between the groups.^33^ Models included the randomization group, 2-week baseline period (period 1) vs 2-week intervention (period 2) and their interaction, and random effects for each participant. The treatment effect (95% CI) was estimated by the treatment group and period interaction, which is equivalent to testing the difference in change from baseline (period 2 minus period 1) in the sleep extension group vs the control group. To confirm the robustness of primary findings, we fit additional models using the analysis of covariance approach with the period 2 value as the dependent variable, treatment group as the independent variable, and period 1 value as covariates.

In secondary analyses, mixed models that adjusted for sex or menstrual cycle were also fit; these covariates were chosen because of the known influence of menstrual cycle on short-term changes in weight. A Pearson correlation coefficient was calculated to assess the relationships between the changes from baseline in sleep duration and the changes from baseline in energy intake. No adjustments were made to *P* values or CIs for multiple comparisons. Baseline characteristics of participants with complete data were compared with those of participants with incomplete data using unpaired, 2-tailed *t* tests and Fisher exact tests. No imputation for missing values was performed.

## Results

Of the 210 adults who provided consent and were assessed for eligibility, 81 were randomized (41 to the control group and 40 to the sleep extension group) initially (Figure 1). One participant in the control group revealed adhering to a weight loss regimen and thus did not meet the study inclusion criteria and was deemed ineligible after randomization.^34^ The 80 participants had a mean (SD) age of 29.8 (5.1) years and consisted of 41 men (51.3%) and 39 women (48.7%). Baseline characteristics of participants were similar between randomization groups (Table 1). None of the participants were using any antihypertensive or lipid-lowering agents or any prescription medication that can affect sleep or metabolism.

**Table 1.** Baseline Characteristics of Participants by Randomization Group

Characteristic	No. (%)	
	Control group (n = 40)	Sleep extension group (n = 40)
Age, mean (SD), y	30.3 (5.5)	29.3 (4.7)
Sex		
Male	21 (52.5)	20 (50.0)
Female	19 (47.5)	20 (50.0)
BMI, mean (SD)	28.1 (1.5)	28.1 (1.3)
Race and ethnicity^a^		
Asian	3 (7.5)	1 (2.5)
Black or African American	9 (22.5)	11 (27.5)
Hispanic	3 (7.5)	3 (7.5)
White	21 (52.5)	20 (50.0)
>1 Race and ethnicity	4 (10.0)	5 (12.5)
Employment status		
Employed		
Full-time	26 (65.0)	23 (57.5)
Part-time	3 (7.5)	3 (7.5)
Student	5 (12.5)	8 (20.0)
Working from home	3 (7.5)	4 (10.0)
Unemployed	3 (7.5)	2 (5.0)
MEQ score, mean (SD)^b^	51.7 (7.4)	50.8 (7.7)
CES-D score, mean (SD)^c^	5.1 (4.7)	6.2 (5.7)
TFEQ score, mean (SD)^d^		
Cognitive restraint	8.9 (2.7)	8.8 (3.7)
Disinhibition	4.9 (3.0)	5.7 (3.3)
Hunger	4.0 (2.7)	4.1 (2.8)
Regular exercise^e^	20 (50.0)	18 (45.0)
Habitual sleep duration, mean (SD), h^f^	6.0 (0.5)	5.9 (0.6)

Abbreviations: BMI, body mass index (calculated as weight in kilograms divided by height in meters squared); CES-D, Center for Epidemiologic Studies Depression scale; MEQ, Morningness-Eveningness Questionnaire; TFEQ, Three-Factor Eating Questionnaire.

^a^ Race and ethnicity data were self-reported.

^b^ The score on the 19-item measure ranges from 16 to 86; scores of 16 to 41 indicate evening type (definitely or moderately evening type), scores of 59 to 86 indicate morning type (definitely or moderately morning type), and scores of 42 to 58 indicate intermediate or neither type.

^c^ The score on the 20-item measure ranges from 0 to 60; scores of 16 or higher indicate a higher frequency of depressive symptoms.

^d^ The score on the 51-item measure ranges from 0 to 20 for Factor I, 0 to 16 for Factor II, and 0 to 15 for Factor III, with higher scores indicating greater levels of restraint, disinhibition, and perceived hunger, respectively.

^e^ Regular exercise was self-reported and defined as engaging in exercise more than twice in a regular week and accumulating at least 90 minutes of moderate or 40 minutes of vigorous exercise.

^f^ Habitual sleep duration was from 1-week wrist actigraphy monitoring during screening.

**Figure 2.**
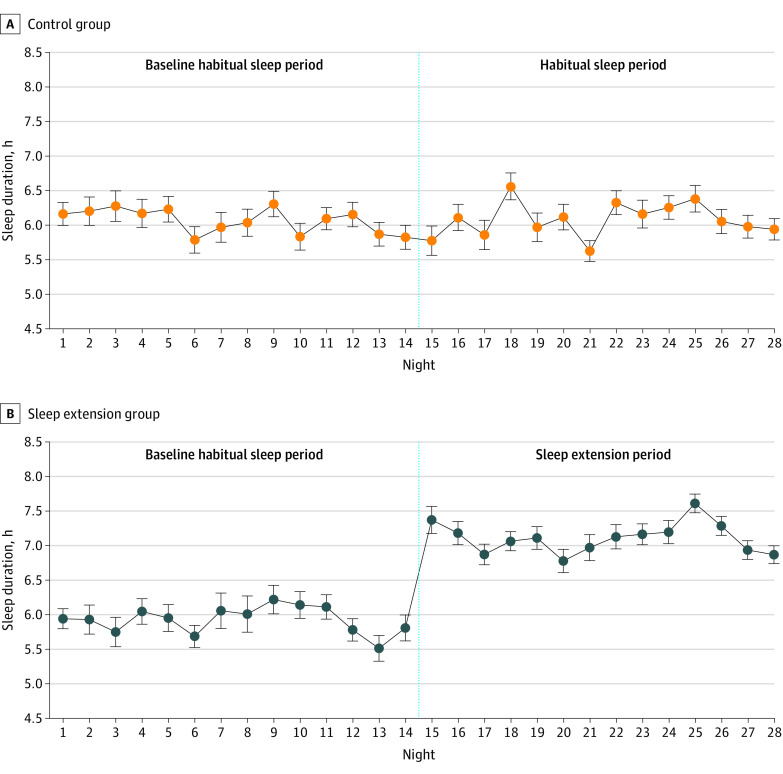
Mean Nightly Sleep Duration by Wrist Actigraphy in Control and Sleep Extension Groups Participants continued to live in their home environment without any prescribed diet or physical activity during the 28 consecutive days of the study. Error bars are SEs of the mean. The vertical dashed line separates the two 2-week sleep periods.

Figure 2 illustrates the mean nightly sleep duration by actigraphy in each group throughout the 4-week study. Participants in the sleep extension group had a significant increase from baseline in mean sleep duration by actigraphy compared with those in the control group (1.2 hours; 95% CI, 1.0-1.4 hours; *P* < .001). The findings were similar with regard to change in sleep duration when only participants' workdays (1.3 hours; 95% CI, 1.0-1.5 hours; *P* < .001) or free days (1.1 hours; 95% CI, 0.7-1.5 hours; *P* < .001) were considered (eTable 1 in Supplement 2). No difference was found in change in sleep efficiency (percentage of time spent asleep during time in bed) between the 2 groups (–0.6 hours; 95% CI, –2.1 to 1.0 hours; *P* = .48), confirming the success of the intervention (eTable 2 in Supplement 2).

**Figure 3.**
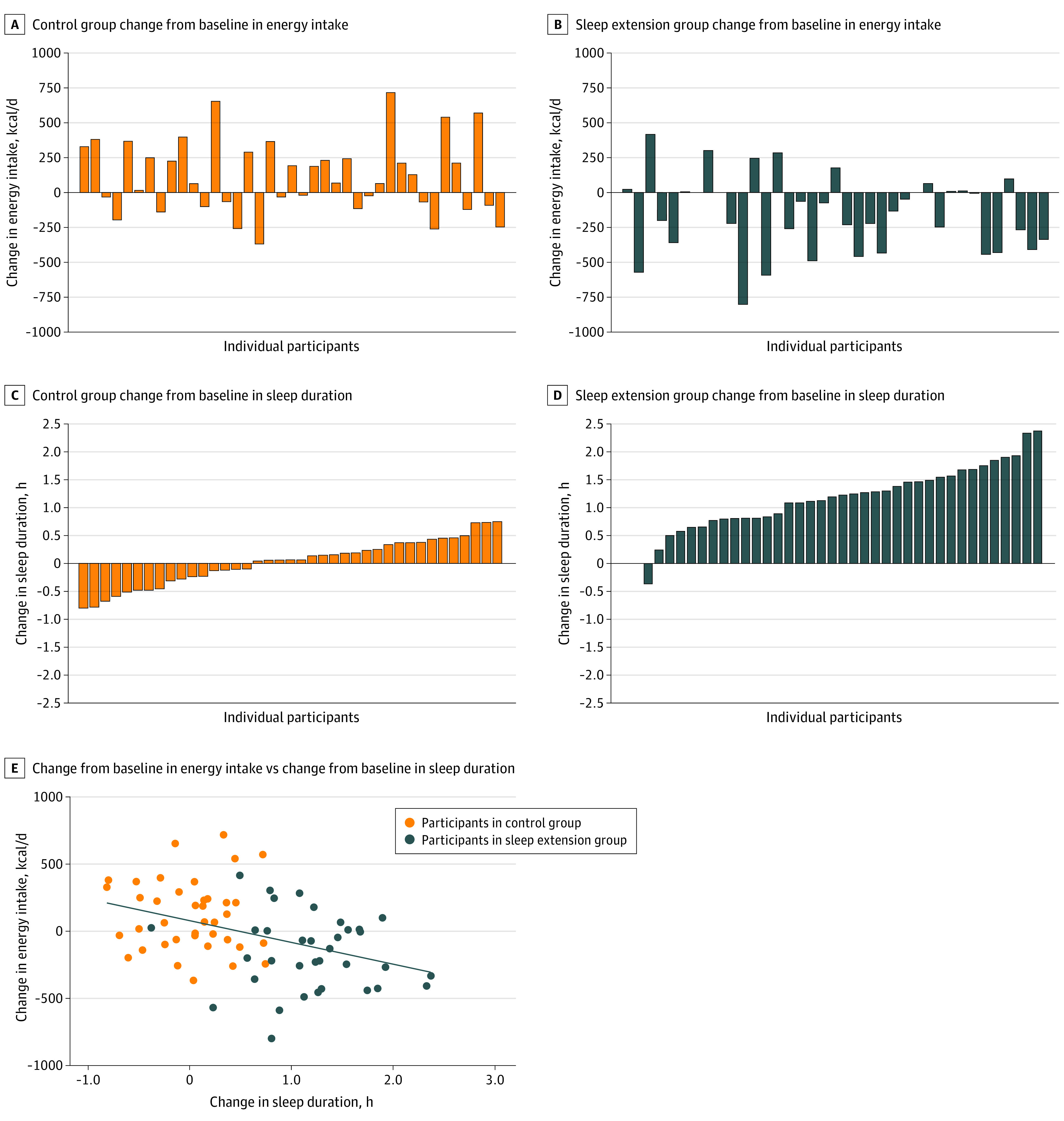
Change From Baseline in Sleep Duration and Energy Intake in Individual Participants A-D, Data are in ascending order of change in sleep duration for the control group and sleep extension group. E, Data were from 74 participants. All available data were used. The line represents the line of best fit from the linear regression model. One participant in the control group and 3 participants in the sleep extension group had missing data in change in sleep duration (ie, missing mean data in at least 1 of 2 study periods). One participant in the control group and 4 participants in the sleep extension group had missing data in change in energy intake. Overall, 1 participant in the control group and 5 participants in the sleep extension group had missing data in either change in sleep duration or change in energy intake.

Energy intake was statistically significantly decreased in the sleep extension group compared with the control group (−270.4 kcal/d; 95% CI, −393.4 to −147.4 kcal/d; *P* < .001). Figure 3A through D illustrates the changes from baseline in energy intake and the changes from baseline in sleep duration in individual participants. There was a significant increase in energy intake from baseline in the control group (114.9 kcal/d; 95% CI, 29.6 to 200.2 kcal/d) and a significant decrease in energy intake from baseline in the sleep extension group (−155.5 kcal/d; 95% CI, −244.1 to −66.9 kcal/d) (Table 2). Considering all participants, the change in sleep duration was inversely correlated with the change in energy intake (*r* = −0.41; 95% CI, −0.59 to −0.20; *P* < .001) (Figure 3E). Each 1-hour increase in sleep duration was associated with a decrease in energy intake of approximately 162 kcal/d (−162.3 kcal/d; 95% CI, −246.8 to −77.7 kcal/d; *P* < .001).

**Table 2.** Effect of Treatment on Energy Intake, Energy Expenditure, and Weight^a^

Variable	Mean (95% CI)							*P* value
	Control group (n = 40)			Sleep extension group (n = 40)			Differences in changes (95% CI)	
	Baseline habitual sleep	Habitual sleep	Change from baseline	Baseline habitual sleep	Sleep extension	Change from baseline		
Energy intake, kcal/d	2665.0 (2468.4 to 2861.6)	2779.9 (2583.3 to 2976.5)	114.9 (29.6 to 200.2)	2976.6 (2782.5 to 3170.7)	2821.1 (2625.1 to 3017.2)	−155.5 (−244.1 to −66.9)	−270.4 (−393.4 to −147.4)	<.001
Total energy expenditure, kcal/d	2693.5 (2499.9 to 2887.1)	2700.7 (2507.1 to 2894.3)	7.2 (−49.0 to 63.3)	2871.1 (2677.6 to 3064.7)	2824.4 (2630.2 to 3018.6)	−46.7 (−105.1 to 11.6)	−53.9 (−134.9 to 27.1)	.19
Energy balance, kcal/d	−0.6 (−69.9 to 68.8)	105.4 (36.0 to 174.8)	106.0 (19.0 to 193.0)	105.4 (36.9 to 174.0)	0.4 (−71.7 to 72.4)	−105.1 (−193.8 to −16.3)	−211.1 (−335.4 to −86.8)	.001
Resting metabolic rate, kcal/d								
Fasting	1563.6 (1487.3 to 1640.0)	1581.1 (1504.5 to 1657.6)	17.4 (−4.9 to 39.7)	1667.8 (1591.3 to 1744.2)	1652.7 (1576.2 to 1729.3)	−15.1 (−38.0 to 7.9)	−32.5 (−64.5 to −0.5)	.047
Postprandial	1837.9 (1746.6 to 1929.2)	1859.7 (1768.0 to 1951.4)	21.8 (−6.5 to 50.1)	1944.9 (1853.4 to 2036.5)	1949.8 (1858.1 to 2041.4)	4.8 (−24.2 to 33.9)	−17.0 (−57.5 to 23.6)	.41
Thermic effect of meal, kcal^b^	45.7 (41.4 to 50.1)	46.6 (42.2 to 51.1)	0.9 (−3.2 to 5.0)	46.3 (41.9 to 50.8)	49.1 (44.6 to 53.6)	2.8 (−1.5 to 7.0)	1.8 (−4.0 to 7.7)	.54
Activity energy expenditure, kcal/d	878.9 (759.7 to 998.0)	863.7 (743.2 to 984.1)	−15.2 (−81.0 to 50.5)	951.2 (832.8 to 1069.7)	918.1 (798.8 to 1037.5)	−33.1 (−100.5 to 34.3)	−17.9 (−112.1 to 76.3)	.71
Activity energy expenditure, kcal/wake hour	51.3 (44.3 to 58.4)	50.7 (43.6 to 57.7)	−0.7 (−4.6 to 3.2)	55.1 (48.1 to 62.1)	57.9 (50.9 to 65.0)	2.9 (−1.1 to 6.8)	3.6 (−2.0 to 9.1)	.21
Weight change, kg^c^	−0.04 (−0.33 to 0.25)	0.35 (0.06 to 0.64)	0.39 (0.02 to 0.76)	0.45 (0.16 to 0.73)	−0.03 (−0.33 to 0.27)	−0.48 (−0.85 to −0.11)	−0.87 (−1.39 to −0.35)	.001
Fat-free mass change, kg	−0.05 (−0.27 to 0.17)	0.22 (−0.003 to 0.44)	0.26 (−0.01 to 0.54)	0.32 (0.11 to 0.54)	−0.03 (−0.26 to 0.19)	−0.36 (−0.64 to −0.08)	−0.62 (−1.02 to −0.23)	.002
Fat mass change, kg	0.004 (−0.08 to 0.09)	0.13 (0.05 to 0.21)	0.13 (0.03 to 0.23)	0.12 (0.04 to 0.20)	−0.00 (−0.08 to 0.08)	−0.12 (−0.22 to −0.02)	−0.25 (−0.39 to −0.10)	.001

^a^ Data were from model-derived estimates and reported as mean (95% CI). *P* values for the differences in changes (sleep extension group minus control group) were from the test of the randomization group by period interaction using linear mixed-effects model approach. All available data were used in the analyses. Energy balance was calculated as the energy intake minus total energy expenditure.

^b^ Calculated from a breakfast meal.

^c^ Weight change represents mean change over each 2-week period using each participant’s slope of daily home weights. In confirmatory analyses, an interrupted time-series approach yielded similar results for weight change. Fat mass and fat-free mass changes were derived from dual-energy x-ray absorptiometry scans that were performed at the beginning and end of each period.

No statistically significant treatment effect was found in total energy expenditure or other measures of energy expenditure (Table 2). Participants in the sleep extension group had a statistically significant reduction in weight compared with those in the control group (−0.87 kg; 95% CI, −1.39 to −0.35 kg; *P* = .001). There was weight gain from baseline in the control group (0.39 kg; 95% CI, 0.02 to 0.76 kg) and weight reduction from baseline in the sleep extension group (−0.48 kg; 95% CI, −0.85 to −0.11 kg) (Table 2).

The findings on energy intake, energy expenditure, and weight were similar after adjustment for the effects of sex or menstrual cycle. No statistically significant differences in baseline characteristics were found between the 75 participants (93.8%) who had complete data on energy intake (primary outcome) vs participants with missing data on energy intake. The proportion of participants with complete data on energy intake was not significantly different between the sleep extension and control groups (90.0% vs 97.5%; *P* = .36). When all reported outcomes were considered, no significant differences (except for depressive symptoms) in baseline characteristics were found between participants with complete data and participants with incomplete or missing data (eTable 3 in Supplement 2). The proportion of participants with complete data on all reported outcomes was similar between the sleep extension and control groups (82.5% vs 85.0%; *P* > .99).

## Discussion

In this RCT of adults with overweight who habitually curtailed their sleep duration, sleep extension reduced energy intake and resulted in a negative energy balance (ie, energy intake that is less than energy expenditure) in real-life settings. To our knowledge, this study provides the first evidence of the beneficial effects of extending sleep to a healthy duration on objectively assessed energy intake and body weight in participants who continued to live in their home environment. Modest lifestyle changes in energy intake or expenditure are increasingly promoted as viable interventions to reverse obesity.

According to the Hall dynamic prediction model, a decrease in energy intake of approximately 270 kcal/d, which we observed after short-term sleep extension, would predict an approximately 12-kg weight loss over 3 years if the effects were sustained over a long term.^14,15^ However, this study cannot infer how long healthy sleep habits may be sustained. Nevertheless, these modeling predictions on weight change suggest that continued adequate sleep duration and beneficial effect on energy intake could translate into clinically meaningful weight loss and help reverse or prevent obesity. Thus, the findings of this study may have important public health implications for weight management and policy recommendations.

The findings of decreased energy intake, negative energy balance, and weight reduction resulting from sleep extension are in agreement with the findings of short-term laboratory sleep-restriction studies showing increased energy intake and weight gain^17^ as well as the findings of prospective epidemiologic studies linking sleep restriction to obesity risk.^8^ A recent meta-analysis of randomized controlled laboratory studies found that short-term sleep restriction over 1 to 14 days of duration in healthy individuals was associated with increases of mean energy intake by approximately 253 kcal/d, as assessed during a single meal.^17^ Another meta-analysis of prospective cohort studies found that the risk of obesity increased by 9% for each 1-hour decrease in sleep duration.^8^ We did not observe a statistically significant change in total energy expenditure by doubly labeled water method or mean daytime activity counts by actigraphy (eTable 2 in Supplement 2). Although some laboratory sleep-restriction studies reported an increase in total energy expenditure of approximately 92 to 111 kcal/d, using a whole-room calorimeter,^35,36^ other studies observed no change.^16,37^ We found a modest reduction in weight after sleep extension, and the composition of weight change was primarily in fat-free mass, which is consistent with the short-term changes in body composition.^38,39^ If sleep is extended over longer periods, weight loss in the form of fat mass would likely increase over time. A few observations suggest that sleeping 7 to 8 hours per night is associated with greater success in weight loss interventions.^40,41,42,43^

In this RCT, we found an overall increase in objective sleep duration of approximately 1.2 hours in participants who habitually slept less than 6.5 hours per night. The change in sleep duration from baseline varied between participants and from night to night in the real-life setting. Overall, the sleep extension group compared with the control group had significantly higher subjective scores in obtaining sufficient sleep, with more daytime energy and alertness and better mood (eTable 4 in Supplement 2). Similar to a previous study of sleep extension,^22^ the present RCT used an individualized counseling approach. Another study used bedtime extension in habitual short sleepers in real-life conditions but obtained variable benefits on sleep, likely because of a lack of an individualized approach or appropriate blinding.^44^ None of these previous studies objectively measured energy intake.

Future similarly rigorous intervention studies of longer duration and using objective assessments of energy balance under real-life conditions are warranted to elucidate the underlying mechanisms and to investigate whether sleep extension could be an effective, scalable strategy for reversing obesity in diverse populations. Along with a healthy diet and regular physical activity, healthy sleep habits should be integrated into public messages to help reduce the risk of obesity and related comorbidities.

### Strengths and Limitations

This study has several strengths. The major strengths are the randomized design and the objective tracking of energy intake and sleep in real-life settings. Most epidemiologic studies linking short sleep duration to body weight relied on self-reported dietary intake.^45^ We did not collect self-reported dietary data because this method is subject to bias and has been shown to be inaccurate compared with the doubly labeled water method.^46,47^ Most experimental studies that measured energy intake used a single meal under unnatural laboratory conditions. We used a validated method to objectively track energy intake by the doubly labeled water method and change in energy stores.^23,48,49^ In this trial, we objectively quantified energy intake after sleep extension while individuals continued their daily routine in their usual environment. Participant blinding and use of actigraphy allowed us to capture true habitual sleep patterns at baseline.^22,50^ In addition, we excluded insomnia and sleep apnea.

This study also has several limitations. We enrolled adults with overweight and used selective eligibility criteria, which may limit generalizability to more diverse populations. The increase in energy intake and weight from baseline that we observed in the control group may have contributed to the significant treatment effects. However, in RCTs, performing a between-group comparison, rather than separate tests against baseline within the groups, is strongly recommended.^51^ The study did not provide information on how long healthy sleep habits could be maintained over longer periods.^44^ We did not systematically assess the factors that may have influenced sleep behavior, but limiting the use of electronic devices appeared to be a key intervention among the participants (eTable 4 in Supplement 2). The doubly labeled water method has a precision of 5%, which may translate into some degree of uncertainty in the energy intake calculations. Although whole-room calorimeters can measure energy expenditure with a higher precision of approximately 1% to 2%, they do not represent real-life measurement and are not feasible over longer periods. We did not assess the underlying biological mechanisms of food frequency and the circadian timing of food intake. Multiple interrelated factors could contribute to the finding of decreased energy intake after sleep extension.^6,52^ Evidence from laboratory sleep restriction studies suggests that increased hunger, alterations in appetite-regulating hormones, and changes in brain regions related to reward-seeking behavior are potential mechanisms that promote overeating after sleep restriction.^6,45^

## Conclusions

This RCT found that short-term sleep extension reduced objectively measured energy intake and resulted in a negative energy balance in real-life settings in adults with overweight who habitually curtailed their sleep duration. The findings highlighted the importance of improving and maintaining adequate sleep duration as a public health target for obesity prevention and increasing awareness about the benefits of adequate sleep duration for healthy weight maintenance.

## Notes

Supplement 1. Trial Protocol

Supplement 2. **eMethods.** Participants, Inclusion and Exclusion Criteria
eReferences
**eTable 1.** Effect of Treatment on Actigraphy-Based Time in Bed and Sleep Duration on All Days, Workdays and Free Days **eTable 2.** Effect of Treatment on Actigraphy-Based Outcomes **eTable 3.** Baseline Characteristics of Participants With Complete vs Incomplete Data **eTable 4.** Self-Reported Outcomes by Visual Analog Scales

Supplement 3.
Data Sharing Statement
