# Supplementary material for: Effect of Sleep Extension on Objectively Assessed Energy Intake Among Adults With Overweight in Real-life Settings: A Randomized Clinical Trial
Source: JAMA Intern Med. 2022 Feb 7;182(4):365–74. doi: 10.1001/jamainternmed.2021.8098 (PMC8822469; doi:10.1001/jamainternmed.2021.8098)
Supplement: Supplement 1. — Trial Protocol [file jamainternmed-e218098-s001.pdf]

PROTOCOL TITLE: HOME SLEEP AND METABOLISM

Purpose and Hypothesis

The overall purpose of this study is to investigate the effects of sleep extension on glucose metabolism and energy balance in habitual short sleepers, while they live in their normal environment. The specific aims are to test the hypotheses that: 1) sleep extension will improve insulin sensitivity and beta cell function; 2) sleep extension will reduce average daily energy intake; 3) sleep extension will increase total daily energy expenditure, mainly due to an increase in physical activity. We further hypothesize that the amount of sleep extension (as quantified by continuous actigraphy monitoring) will predict the magnitude of the beneficial effects on glucose metabolism, energy intake and energy expenditure.

Potential Benefits of the proposed research to the subjects and others

The information gained from these studies may provide novel and important insights to our understanding of the processes underlying the relationships between sleep and energy metabolism. Furthermore, the findings are expected to provide key data needed for the design of future large-scale clinical trials that will determine whether improving sleep duration could be a novel behavioral strategy to counteract the current epidemics of diabetes and obesity. If the proposed study demonstrates a clear benefit of sleep extension, these findings could be translated into clinical practice such that it may provide a strong incentive to millions of Americans to implement “healthy sleep habits” as part of traditional lifestyle regimens aiming to prevent or treat diabetes and obesity. The risks are only minimal and the risk-to-benefit ratio is very small.

Methodology

Study protocol

This is a randomized controlled parallel-group study in overweight adults (age: 21-40 years and BMI=25.0-29.9 kg/m^2) who habitually sleep on average 6.5 hours or less per night (as confirmed by actigraphy). After 2-weeks of habitual sleep [baseline] period, subjects will be randomized to either 2-weeks of sleep extension (Extension group, n=40) or 2-weeks of continued habitual sleep (Control group, n=40). We will perform the same procedures during each 2-week sleep period and changes from baseline will be compared between the 2 groups. Randomization assignment will be prepared by a statistician prior to the study (with gender as the stratification factor to ensure balance between groups) and be kept in sealed envelopes until subjects complete the first 2-week habitual sleep [baseline] period (i.e. until Day15).

The subjects will be blinded to randomization and sleep extension in order to effectively capture habitual sleep patterns. This blinding is necessary based on a large randomized controlled trial at NIH/NIDDK (led by Dr.Cizza) showing that if the subjects know about the importance of sleep and sleep extension intervention when they start the study, they begin extending their sleep before the sleep intervention, which modifies the "true" study baseline (Cizza et al. Plos One 2014). We will debrief the habitual sleep group at the end of the study. A debriefing script for habitual sleep group will fully explain the 2 sleep groups and the subjects will be told which group they were randomized. The sleep extension group will be debriefed at the end of 2-week baseline period with a separate debriefing script. All subjects will be provided with information about the beneficial effects of good sleep habits for health at the end of the study.

Sleep-wake patterns will be continuously monitored throughout the protocol by actiwatch. Body composition will be measured by dual-energy X-ray absorptiometry (DXA) in the morning of days 1 and 15, and in the morning following the day 28. On the first morning of each 2-week sleep period, the subjects will provide baseline urine sample before they ingest 18O- and 2H-labeled water and urine samples (at 1h, 2h, 3h and 4h) after dosing with labeled water will be collected. On last morning of each 2-week sleep period, two more urine samples will be collected and resting metabolic rate will be measured under basal conditions (indirect calorimetry). Subjects will then undergo a mixed meal tolerance test (MTT) during which they will consume a standardized liquid meal and their metabolic rate will be simultaneously measured by indirect calorimetry under resting conditions for the

next 4 hours in the postprandial state for assessments of thermic effect of meal. Blood samples will be collected during MTT for measurements of glucose, insulin and c-peptide. Anthropometric measurements including body weight and height will be performed in the CRC prior to metabolic testing in the morning; and subjects will measure and record their body weight every morning at home, using calibrated scales provided by the investigators. On certain days during the study, they will also complete visual analog scales for appetite and sleepiness before and after main meals.

During the first 2-week baseline period, all subjects will be asked to follow their habitual bedtimes at home. On Day 15, subjects who are randomized to the extension group will begin the sleep extension intervention and receive sleep hygiene recommendations. Actiwatch data from the first 2-week [baseline] period will be reviewed in order to accommodate the extended bedtimes in subject's daily routine in the best possible way. Subjects will be asked to follow individually designed bedtimes aiming to increase the bedtime duration to 8.5 hours per night, taking into account personal schedules and priorities. On Day 22, actigraphy data from the first week of the sleep extension intervention will be reviewed and further adjustments on bedtimes will be done, as needed. Subjects who are randomized to control group will be told to continue their habitual sleep habits until the end of the study. Control group will also meet with the investigators (but will not receive sleep hygiene recommendations) on Day15 and Day 22 to review the actiwatch data and to balance the amount of time spent with subjects in each group.

Resting metabolic rate and thermic effect of meal by indirect calorimery

Resting metabolic rate will be measured under basal conditions after a 12-hour fast for 40 minutes. The metabolic system will be calibrated before each measurement using a mixed gas tank of 5% CO_2, 21% O_2 and balance nitrogen and a single tank of nitrogen only. This portable metabolic system measures the concentration of oxygen and carbon dioxide in air streams entering and exiting a clear plastic hood (canopy) placed over the subject’s head. Subject will be asked to remain in bed (awake and motionless) for the entire duration of the procedure. After a 10-minute period of habituation, respiratory gas exchange will be measured and averaged over 30 minutes to determine the resting metabolic rate. Measurements will be performed under continuous monitoring of our trained personnel of the CRC metabolic kitchen under the supervision of the Bionutrition Research Manager.

After the subjects ingest the standardized liquid mixed meal, they will then return to a resting position in bed and their metabolic rate will be measured by indirect calorimetry (simultaneously with blood sampling) for the next 4 hours in the postprandial state to assess thermic effect of meal. The meal will provide 54% of the energy from carbohydrate, 21% from fat, and 25% from protein. Blood samples will be time marked during metabolic rate measurements and artifacts due to blood sampling will be excluded, as necessary. Blood samples will be collected at -10, -5, 0, 10, 20, 30, 60, 90, 120, 150, 180 and 240 min. Indices of insulin sensitivity, beta cell responsiveness, hepatic insulin extraction, and disposition index will be derived using the oral minimal model [1]. The area under the 4-h postprandial curve minus the resting metabolic rate will be divided by the caloric content of meal and multiplied by 100 to obtain an estimate of the thermic effect of meal (in % of energy intake).

Doubly labeled water (DLW) method

This method uses two stable isotopic tracers safe for use in humans. The principle of the method is that after a loading dose of water labeled with deuterium, a stable isotope of hydrogen, and the stable isotope ^{18}O, these tracers quickly equilibrate in body water. The deuterium is eliminated from the body as water and the elimination rate is thus proportional to water turnover. The ^{18}O is eliminated as water and carbon dioxide and thus its elimination is proportional to the sum of water turnover and carbon dioxide production. The difference between these two elimination rates is, therefore, proportional to carbon dioxide production. Total energy expenditure can be calculated from carbon dioxide production using common indirect calorimetric equations. The tracer elimination rates will be determined using equations of Schoeller et al. [2] from urines collected on the day the tracer is given by mouth at the beginning of each 2-week sleep period and again at the end of each 2-week

period. The DLW method has a coefficient of variance of ±1% as determined in multiple laboratories and has been validated in various subject populations [2-5].

Body weight and body composition measurements

Body weight will be determined by the mean of two consecutive measurements obtained in the morning following an overnight fast and morning void. Whole body fat percentage will be measured using DXA. Body fat will be determined by multiplying body weight by the percentage of body fat from the DXA. The bone mineral content by DXA and body fat will be subtracted from body weight to determine fat-free mass. We have chosen DXA to measure body composition because it is the most reproducible procedure and thus provides us the highest power for a change in energy stores [6], and early phase body composition changes have been previously quantified by DXA using sample sizes similar to that of the proposed studies [*reviewed in* [7]].

Questionnaires

All eligible subjects will complete standard questionnaires (one time at baseline on Day 1) to capture their usual dietary habits (Three Factor Eating Questionnaire-TFEQ [8]), sleep quality (Pittsburgh Sleep Quality Index- PSQI [9]), choronotype (Morningness-Eveningness Questionnaire-MEQ [10]) and mood (Center for Epidemiologic Studies of Depression-CESD [11]).

Statistical analysis

We propose to complete the study in 80 overweight young individuals (extension group [n=40] and control group [n=40]) using a randomized, controlled parallel-group design. We will obtain the same assessments during each 2-week sleep period (i.e. 2-week baseline and 2-week intervention periods) and changes from baseline will be compared between the extension and control groups. Mean (SD) values for all continuous variables and frequency distributions for all categorical variables will be used for descriptive analyses. Normality will be tested for all continuous variables. If there is evidence of non-normality, then a transformation such as the logarithm will be used. We will assess between group differences using a two-sided two-sample t-test at the significance level of 0.05. The nonparametric Wilcoxon rank-sum test will only be used if data transformation is not successful. Subjects' changes between the two sleep periods will be plotted to further explore inter-individual variability. We plan to account for dropouts as follows. Demographic and health characteristics of the subjects who have completed the study will be compared to those of the dropouts to assess for any evidence of bias. In addition to the analysis in those who competed the entire study (upon which the power calculations were conservatively based), analyses using all subjects with imputation of the missing data will be performed to ensure that findings are robust. Several imputation methods will be considered including multiple imputations or last observation carried forward as well as assuming that dropouts will have the worst/best outcome.

In secondary analyses, we will test the hypothesis that the amount of sleep extension will predict the magnitude of the beneficial effects on glucose metabolism, energy intake and energy expenditure. Average sleep duration in each 2-week period will be measured using the actiwatch [12-14]. For each subject, the average change in sleep duration will be calculated by subtracting the average sleep duration during the 2-week habitual sleep [baseline] period from the average sleep duration during the 2-week intervention period. Pearson correlation coefficients will be calculated, along with corresponding 95% confidence intervals, to assess the strength of the association, and linear regression models (with change in insulin sensitivity, energy intake and total energy expenditure as the dependent variable and the average increase in sleep duration as the independent variable) will be fit to get estimates of the magnitude of the effect.

In order to achieve a final sample size of 80 subjects, we anticipate that a total of 210 subjects will need to be enrolled over 5-year period as follows. We estimate that about one third of the 210 subjects (n=70) will have sleep apnea or other sleep disorders by polysomnography, and about 20% of the remaining 140 subjects (n=30) will show abnormal findings on screening OGTT and routine blood tests, and thus be excluded. Based on our new preliminary study, we estimate that about 10% of the remaining 110 subjects (n=10) will drop out due to poor compliance with the actiwatch. Based

on our previous experience with protocols involving similar study duration and design, we estimate that about 20% of the remaining 100 subjects (n=20) will drop out during the actual 28-day protocol, and thus a total number of n=80 subjects will complete the study.

Human Subjects and Protection from Risk:

<u>Human Subjects Involvement and Characteristics:</u>

There will be no selection criteria based on gender, race or ethnic background. We will not recruit from vulnerable populations, such as pregnant women, prisoners, institutionalized individuals, or others who may be considered vulnerable populations. We will recruit 250 subjects to have a final study sample of n=80. We estimate that 50% of the subjects will be women.

<u>Research Material:</u>

Research material will be blood samples for metabolic and hormonal measurements, polysomnographic sleep recordings, data on activity and sleep-wake schedule from the actiwatch and, energy expenditure and intake, resting metabolic rate and thermic effect of meal, body composition measurements, and food diaries and visual analog scales for appetite and sleepiness. All material will be used only for research purposes and subject confidentiality will be protected according to guidelines. The data will be collected, stored, and managed in full compliance with HIPAA regulations as well as according to regulations by the IRB at the University of Chicago.

<u>Potential Risks:</u>

There may be minimal discomfort associated with venipuncture and slight bruising at the site that will recover in a short period of time. There is also slight risk of inflammation of the vein or infection. However, our nurses who perform these procedures are highly trained and thus these risks associated with intravenous puncture and blood withdrawals are minimal. The amount of blood withdrawn is minimized to prevent significant blood loss. Baseline hematocrit will be measured in all subjects to ensure that the risk is not excessive. Minor temporary skin irritation could occur with the surface electrodes or sensors used for screening sleep recordings and activity monitors. Dual-energy X-ray absorptiometry (DXA) scan is a noninvasive procedure and the amount of radiation exposure is extremely low (less than one-tenth of the dose received during a standard chest x-ray). Indirect calorimetry is a non-invasive measure of resting energy expenditure in which a clear plastic hood is placed over the head and shoulders for 40 minutes, during which time the subject can watch TV and relax. The hood may lead to feelings of claustrophobia. Doubly labeled water method involves the use of water containing two stable isotopic tracers, ^{2}H (deuterium) and ^{18}O. These two tracers are not radioactive and do not expose the subjects to any ionizing radiation. The method has been used to measure total energy expenditure and body water in thousands of subjects including neonates, pregnant and lactating women, children, and adults in many different state of health and disease (Schoeller DA.J Nutr,1999). Dr.Schoeller's laboratory alone has collaborated on over 3,000 such studies. To our knowledge, no serious adverse events have been reported. The safety of the use of doubly labeled water have been summarized in a user's manual prepared by the International Atomic Energy Agency beginning (Schoeller DA et al, IAEA page 3. Human Health Series No. 3, Vienna, 2009). There are no other known risks involved in any of the other measurement procedures.

<u>Procedures For Protecting Against Risk:</u>

Every effort will be made by all study personnel to protect subjects' safety and minimize risk. The risks associated with blood drawing will be minimized by using sterile techniques, close supervision and care of all catheter sites, and by applying pressure to the puncture site after removal of the needle or catheter. A cooling or heating apparatus will be available in the event that local venipuncture site swelling develops. The amount of blood withdrawn will be minimized in accordance with the University of Chicago Institutional Review Board regulations. Baseline hematocrit will be measured in all subjects to ensure that the risk is not excessive. A nutritionist will be present during indirect calorimetric testing and we will exclude subjects with claustrophobia. A physician will be in the immediate vicinity during all invasive procedures. If a subject discloses the intent to harm himself or

herself, the immediacy of the situation will be determined and the subject will be referred for psychiatric counseling or taken to a treatment facility. In the event of physical injury resulting from this research, the University of Chicago Hospitals will provide free emergency medical care. Confidentiality of the subjects will be preserved.

Inclusion of Women and Minorities

The proposed studies will be open to enrollment to both women and men. We proposed to complete the study in 80 subjects and we estimate that 50% will be women. There will be no selection criteria based on race or ethnic background. Based on our local demographic as well as the estimated prevalence of short sleep duration in the general population, it is anticipated that minority subjects will be well represented in the proposed studies.

Inclusion of Children

Children will not be enrolled in the proposed studies. Although there is epidemiologic evidence for an association with short sleep duration and obesity risk in children, there are no sufficient data from well-controlled laboratory studies in children to justify the inclusion of children in this project. Moreover, children have a different physiology for sleep need than adult population, therefore studying children remains beyond the focus of the current proposal and the minimum age requirement will be 21 years.

Data & Safety Monitoring Plan:

Prior to initiation of the study, the University of Chicago Institutional Review Board (IRB) will review and approve the protocol. The IRB will continue to review the progress of the protocol at least annually following initial approval. IRB requires that the investigators report enrollment and dropout rates, any protocol deviations, any adverse events (expected, unexpected and serious) and general progress of the study. The research procedures performed in this study are considered minimal to moderate risk and therefore a formal Data Safety Monitoring Committee will not be necessary per the University of Chicago IRB guidelines. During the protocol procedures that require inpatient or outpatient hospital admission, the subjects will be monitored daily by a physician investigator and nursing staff. As part of the NIH funded Clinical and Translational Science Awards (CTSA) program at the University of Chicago, Gary Toback MD, PhD serves as the Research Subject Advocate. His role is to monitor and ensure the safety of research participants for all protocols that are being conducted at the Clinical Resource Center at the University of Chicago. Dr.Toback will also participate in the process of data and safety monitoring in the proposed studies. Any adverse events will be reported immediately to the investigator for evaluation. Any unanticipated problem will be evaluated by the PI and reported immediately to the IRB if the problem is life threatening, within 10 days if serious, or in the annual continuing review report if it does not affect risk to the subject.

Compensation

Subjects participating in the study will receive $2300 in compensation upon completion of the entire study. Subjects will receive $75 for completing the screening procedures, and $2225 for completing the entire 28-day study phase including 4 half days metabolic testing in the research unit.

Subject Recruitment:

Subjects will be recruited from the local community using flyers and web advertisements. All paper advertisements will indicate a standard email address (sleepstudy@uchicago.edu), which will auto-reply to send to the volunteers a web link of an online pre-screening survey administered under REDCap at the University of Chicago. The web-based advertisement will indicate the web link for volunteers to click on. The REDCap consortium supports a secure web application designed exclusively to support data capture for research studies.

This initial online pre-screening process will help to determine whether volunteers meet the initial eligibility criteria (e.g. age, BMI etc.) and thus will allow the investigators to minimize the number of subjects who will undergo the laboratory screening tests. Study investigators will only contact the

volunteers who meet the initial eligibility criteria to schedule a face-to-face interview in the sleep laboratory for the informed consent process. To contact the volunteers, the investigators will use the email address that the volunteers provided on the online survey. During face-to face interview, the nature of the research project will be described in detail to the subjects and written informed consent will be obtained after a member of the research team has explained all details of the study, and the subject has received satisfactory answers to all of his/her questions.

Overweight men and women (age: 21-40 years; BMI: 25.0 to 29.9 kg/m^2) who habitually sleep on average 6.5 hours or less per night (as confirmed by actigraphy) will be studied. Subjects will be required to have stable sleep habits for the past 6 months. Exclusion criteria will be: obstructive sleep apnea by laboratory polysomnography or history of any other sleep disorder, night or rotating shift work (current or in the past 2 years), habitual daytime naps, recent (< 4 week) travel across time zones, extreme chronotypes, any acute or chronic medical condition, diabetes, prior or current eating or psychiatric disorders, claustrophobia, irregular menstrual periods, menopause, pregnancy, alcohol abuse, excessive caffeine intake, smoking, illegal drug use, subjects who are currently following a weight loss regimen or any other special diet or exercise programs, subjects who have received iv or oral contrast in the past 2 weeks, and abnormal findings on screening blood testing. Subjects will also be required not to take any prescription medication that can affect sleep or metabolism with the exception of antihypertensive and lipid lowering agents as follows. Subjects who are taking anti-hypertensive medication and/or lipid-lowering agents will be included only if they are well controlled and on a stable regimen (no change in medications in the previous 3 months). However, beta-blockers and thiazide diuretics will not be allowed, as these medications are known to affect insulin sensitivity. Women will be required to not be on hormone replacement therapy.

Subjects who meet the eligibility criteria will undergo the following laboratory screening procedures: Step1: an overnight laboratory polysomnography to rule out obstructive sleep apnea and other sleep disorders. Subjects who fail Step1 screening will not continue to Step2. Step2: physical examination, screening laboratory blood tests (complete blood count, complete metabolic panel, lipids, and pregnancy test for women) and a standard morning 2-h OGTT (to exclude diabetes; 1 fasting blood samples and 1 blood sample at 2-hour). Subjects will wear the actiwatch for one week to confirm habitual sleep duration of 6.5 hours or less. Subjects who pass the Step2 screening procedures will be eligible for the protocol.

REFERENCES CITED

1. Cobelli C, Toffolo GM, Dalla Man C, et al. Assessment of beta-cell function in humans, simultaneously with insulin sensitivity and hepatic extraction, from intravenous and oral glucose tests. *Am J Physiol Endocrinol Metab.* 2007;293(1):E1-E15.
2. Schoeller DA. Measurement of energy expenditure in free-living humans by using doubly labeled water. *The Journal of nutrition.* 1988;118(11):1278-1289.
3. Ravussin E, Gautier JF. Metabolic predictors of weight gain. *Int J Obes Relat Metab Disord.* 1999;23 Suppl 1:37-41.
4. Westerterp KR, Brouns F, Saris WH, ten Hoor F. Comparison of doubly labeled water with respirometry at low- and high-activity levels. *J Appl Physiol.* 1988;65(1):53-56.
5. Seale JL, Rumpler WV, Conway JM, Miles CW. Comparison of doubly labeled water, intake-balance, and direct- and indirect-calorimetry methods for measuring energy expenditure in adult men. *The American journal of clinical nutrition.* 1990;52(1):66-71.
6. Heymsfield SB, Pietrobelli A, Wang Z, Saris WH. The end of body composition methodology research? *Current opinion in clinical nutrition and metabolic care.* 2005;8(6):591-594.
7. Heymsfield SB, Thomas D, Nguyen AM, et al. Voluntary weight loss: systematic review of early phase body composition changes. *Obesity reviews : an official journal of the International Association for the Study of Obesity.* 2011;12(5):e348-361.
8. Stunkard AJ, Messick S. The three-factor eating questionnaire to measure dietary restraint, disinhibition and hunger. *J Psychosom Res.* 1985;29(1):71-83.

9. Buysse DJ, Reynolds CF, 3rd, Monk TH, Berman SR, Kupfer DJ. The Pittsburgh Sleep Quality Index: a new instrument for psychiatric practice and research. *Psychiatry Res.* 1989;28(2):193-213.
10. Horne JA, Ostberg O. A self-assessment questionnaire to determine morningness-eveningness in human circadian rhythms. *Int J Chronobiol.* 1976;4(2):97-110.
11. Roberts RE, Vernon SW. The Center for Epidemiologic Studies Depression Scale: its use in a community sample. *The American journal of psychiatry.* 1983;140(1):41-46.
12. Knutson KL, Rathouz PJ, Yan LL, Liu K, Lauderdale DS. Intra-individual daily and yearly variability in actigraphically recorded sleep measures: the CARDIA study. *Sleep.* 2007;30(6):793-796.
13. Laurderdale D, Knutson K, Lijing L, Rathouz PJ, Hulley SB, Liu K. Objectively Measured Sleep Characteristics among Early-Middle-Aged Adults The CARDIA Study. *Am J Epidemiol* 2006;164(1):5-16.
14. Jean-Louis G, von Gizycki H, Zizi F, Spielman A, Hauri P, Taub H. The actigraph data analysis software: I. A novel approach to scoring and interpreting sleep-wake activity. *Percept Mot Skills.* 1997;85(1):207-216.
