# Supplementary material for: Effect of Sleep Extension on Objectively Assessed Energy Intake Among Adults With Overweight in Real-life Settings: A Randomized Clinical Trial
Source: JAMA Intern Med. 2022 Feb 7;182(4):365–74. doi: 10.1001/jamainternmed.2021.8098 (PMC8822469; doi:10.1001/jamainternmed.2021.8098)
Supplement: Supplement 2. — eMethods. Participants, Inclusion and Exclusion Criteria eReferences eTable 1. Effect of Treatment on Actigraphy-Based Time in Bed and Sleep Duration on All Days, Workdays and Free Days eTable 2. Effect of Treatment on Actigraphy-Based Outcomes eTable 3. Baseline Characteristics of Participants With Complete vs Incomplete Data eTable 4. Self-Reported Outcomes by Visual Analog Scales [file jamainternmed-e218098-s002.pdf]

Supplementary Online Content

Tasali E, Wroblewski K, Kahn E, Kilkus J, Schoeller DA. Effect of sleep extension on objectively assessed energy intake among adults with overweight in real-life settings: a randomized clinical trial. *JAMA Intern Med.* Published online February 7, 2022. doi:10.1001/jamainternmed.2021.8098

eMethods. Participants, Inclusion and Exclusion Criteria

eReferences

eTable 1. Effect of Treatment on Actigraphy-Based Time in Bed and Sleep Duration on All Days, Workdays and Free Days

eTable 2. Effect of Treatment on Actigraphy-Based Outcomes

eTable 3. Baseline Characteristics of Participants With Complete vs. Incomplete Data

eTable 4. Self-Reported Outcomes by Visual Analog Scales

This supplementary material has been provided by the authors to give readers additional information about their work.

© 2022 Tasali E. et al. *JAMA Internal Medicine.*

eMethods. Participants, Inclusion and Exclusion Criteria

Adult men and women aged 21 to 40 years who had a body mass index between 25.0 and 29.9 kg/m^2 and an average habitual sleep duration less than 6.5 hours per night were eligible. Participants were required to have stable self-reported sleep habits for the past 6 months. Participants were recruited from the community and completed an initial online pre-screening survey via REDCap™ followed by a face-to-face interview. Those who met the inclusion criteria underwent laboratory screening (polysomnography, oral glucose tolerance test, blood tests) to determine eligibility. Habitual sleep duration was confirmed by a 1-week screening actigraphy at home. Exclusion criteria were: obstructive sleep apnea as assessed by laboratory polysomnography (apnea hypopnea index [AHI] >5), insomnia or history of any other sleep disorder, night or rotating shift work (current or in the past 2 years), regular napping, travel across time zones during the previous 4 weeks, extreme chronotypes by Morningness-Eveningness Questionnaire, any acute or chronic medical conditions, diabetes, prior or current eating or psychiatric disorders, claustrophobia, irregular menstrual periods, menopause, pregnancy, alcohol abuse, excessive caffeine intake, smoking, illegal drug use, subjects who are currently following a weight loss regimen or any other special diet or exercise programs, subjects who have received intravenous or oral contrast material in the past 2 weeks, and abnormal findings on screening blood tests. Subjects were also required not to take any prescription medication that can affect sleep or metabolism except for antihypertensive and lipid lowering agents. Women were not allowed to be on birth control medications or hormone replacement therapy.

Sleep intervention: Individualized Sleep Hygiene Counseling

Sleep-wake patterns were continuously monitored at home by wrist actigraphy. During the 2-week habitual sleep period at baseline, all participants were instructed to continue their daily routine and habitual sleep behaviors. On the morning of day 15, participants met with the study investigator (E.T.) in the research center. Those randomized to extension group received individualized sleep

© 2022 Tasali E. et al. *JAMA Internal Medicine.*

hygiene counseling via a structured, face-to-face interview of approximately 1-hour duration. The overall goal was to accommodate extended bedtimes in participants' lifestyles in the best possible way. First, actigraphy data from baseline period was briefly reviewed with the participant. Next, the key components of sleep hygiene were discussed. Specifically, the habitual sleep-wake schedules on workdays and free days, naps (if any), environmental factors (bedroom temperature, noise, ambient light), bedtime routine, television/electronic use, and physiological factors (e.g., exercise, caffeine) were reviewed. As necessary, factors related to sleep partner, children, other household members and pets were considered. Individual recommendations on sleep hygiene were provided to better implement extended bedtimes into the daily routine. At the end of the interview, participants were provided with individualized bedtime and wake-up time schedules to follow at home for two weeks, aiming to extend bedtime duration to 8.5 h. The schedules were designed by mutual agreement considering personal schedules and priorities. On day 22, participants returned for a brief follow-up visit of approximately 15-min. Actigraphy data from the first intervention week was reviewed and further sleep counseling was provided, as needed.

Doubly Labeled Water Method

We measured total energy expenditure using the doubly labeled water method. [1-3] This method measures the sum of energy expenditure for resting metabolic rate, thermic effect of meals and physical activity. On day 1, participants reported to the research unit in the morning after an overnight fast. Participants completely voided to collect pre-dose urine specimen and two 5 mL aliquots were transferred to o-ring sealed, screw capped Corning cryotubes. Subjects drank a sterile loading dose of 1.8 g 10 AP ^{18}O and 0.12 g 99.9 AP ^{2}H enriched water per kg of estimated total body water. The dose bottle was washed with 50 g of tap water which was consumed by the participants. Subjects voided at 1 hour after the dose and that specimen was discarded.

Participants voided again at 3 hours and 4 hours after the dose and aliquots were collected. Participants fasted throughout the 4-h specimen collection period. They were provided 250 mL

© 2022 Tasali E. et al. *JAMA Internal Medicine.*

water between 1-h and 3-h post dose, and the water consumed was recorded and subtracted from the total body water. Participants returned to the research unit on the morning of day 14 after an overnight fast and two voids were collected 1 hour apart, and aliquots stored. Urine specimens were stored at -20ºC and shipped to the University of Wisconsin-Madison for analysis. The rate of CO_2 production was calculated using Schoeller equation A6 as modified by Racette et al (1994).[2,4] TEE was calculated from rCO_2 as described by Black et al.[5], assuming participants consumed a typical diet with a food quotient of 0.86 individually accounting for the change in mass of protein and fat during each 2-week period.[6] For each 2-week period, the change in body energy stores was computed from the regression (slope, grams/day) of daily home weights and change in body composition i.e., fat mass (FM) and fat free mass (FFM) as measured by dual-energy x- ray absorptiometry.

Resting metabolic rate and thermic effect of meal and activity energy expenditure.

Resting metabolic rate (RMR) was measured by indirect calorimetry using the DeltaTrac (SensorMedix) and Vmax (SensorMedix) metabolic carts. The same unit was used in any given participant during baseline and treatment periods. Participants reported to the research unit in the morning after an overnight fast. After calibration against a standard gas mixture and 30 minutes of resting period in a semi-recumbent position in bed, a clear plastic canopy and flexible plastic seal was placed over participant's head and respiratory gas exchange was measured for 40 minutes. The first 10 minutes of measurements were discarded to collect data under steady state. The subjects then consumed a standardized breakfast (liquid meal) within 5 minutes. Thermic effect of the meal was measured using the same metabolic cart over the next 4 hours using a protocol of 50 min under the canopy and followed by 10 min with the canopy removed in a repeated cycle for participants' comfort. Thermic effect of meal was calculated as previously described.[7] Activity energy expenditure was calculated by subtracting the resting metabolic rate and thermic effect of meal from the total energy expenditure measured by doubly the labeled water method.[7,8]

© 2022 Tasali E. et al. *JAMA Internal Medicine.*

eReferences

1. Schoeller DA. Measurement of energy expenditure in free-living humans by using doubly labeled water. *J Nutr.* 1988;118(11):1278-1289.
2. Schoeller DA, Ravussin E, Schutz Y, Acheson KJ, Baertschi P, Jequier E. Energy expenditure by doubly labeled water: validation in humans and proposed calculation. *Am J Physiol.* 1986;250(5 Pt 2):R823-830.
3. Speakman JR. The history and theory of the doubly labeled water technique. *Am J Clin Nutr.* 1998;68(4):932S-938S.
4. Racette SB, Schoeller DA, Luke AH, Shay K, Hnilicka J, Kushner RF. Relative dilution spaces of 2H- and 18O-labeled water in humans. *Am J Physiol.* 1994;267(4 Pt 1):E585-590.
5. Black AE, Prentice AM, Coward WA. Use of food quotients to predict respiratory quotients for the doubly-labelled water method of measuring energy expenditure. *Hum Nutr Clin Nutr.* 1986;40(5):381-391.
6. Hall KD, Guo J, Chen KY, et al. Methodologic considerations for measuring energy expenditure differences between diets varying in carbohydrate using the doubly labeled water method. *Am J Clin Nutr.* 2019;109(5):1328-1334.
7. Schoeller DA, Shay K, Kushner RF. How much physical activity is needed to minimize weight gain in previously obese women? *Am J Clin Nutr.* 1997;66(3):551-556.
8. Westerterp KR. Exercise, energy expenditure and energy balance, as measured with doubly labelled water. *Proc Nutr Soc.* 2018;77(1):4-10.

© 2022 Tasali E. et al. *JAMA Internal Medicine.*

eTable 1. Effect of Treatment on Actigraphy-Based Time in Bed and Sleep Duration on All Days, Workdays and Free Days

	Control group (n=40)			Extension group (n=40)				
**Variable**	**Baseline habitual sleep**	**Habitual sleep**	**Change from baseline**	**Baseline habitual sleep**	**Sleep extension**	**Change from baseline**	**Differences in changes (95% CI)**	***P* value**
**All days**								
Time in bed, h	6.87 (6.72 to 7.03)	6.91 (6.76 to 7.07)	0.04 (-0.12 to 0.20)	6.74 (6.58 to 6.89)	8.17 (8.01 to 8.32)	1.43 (1.27 to 1.59)	1.39 (1.16 to 1.62)	<0.001
Sleep duration, h	6.05 (5.90 to 6.20)	6.07 (5.92 to 6.22)	0.02 (-0.14 to 0.17)	5.92 (5.77 to 6.07)	7.11 (6.96 to 7.26)	1.19 (1.03 to 1.34)	1.17 (0.95 to 1.39)	<0.001
**Workdays**								
Time in bed, h	6.74 (6.57 to 6.91)	6.79 (6.62 to 6.96)	0.05 (-0.14 to 0.23)	6.63 (6.46 to 6.80)	8.14 (7.96 to 8.31)	1.51 (1.32 to 1.69)	1.46 (1.20 to 1.72)	<0.001
Sleep duration, h	5.95 (5.78 to 6.11)	5.95 (5.79 to 6.12)	0.003 (-0.17 to 0.17)	5.82 (5.66 to 5.99)	7.07 (6.91 to 7.24)	1.25 (1.07 to 1.42)	1.25 (1.00 to 1.49)	<0.001
**Free days**								
Time in bed, h	7.17 (6.93 to 7.41)	7.20 (6.96 to 7.44)	0.03 (-0.26 to 0.31)	6.95 (6.72 to 7.19)	8.29 (8.05 to 8.53)	1.33 (1.05 to 1.62)	1.30 (0.90 to 1.71)	<0.001
Sleep duration, h	6.30 (6.08 to 6.52)	6.34 (6.12 to 6.57)	0.04 (-0.23 to 0.31)	6.12 (5.90 to 6.35)	7.23 (7.01 to 7.46)	1.11 (0.84 to 1.38)	1.07 (0.68 to 1.45)	<0.001

Data were from model-derived estimates and reported as mean (95% CI). *P* values for the differences in changes (extension group minus control group) were from the test of the treatment group by period interaction using a linear mixed-effects model approach. All available data were used in the analyses. Workdays and free days were self-reported. Time in bed (h) is the total time spent in bed between bedtime (the time point when first lying down in bed) and wake-up time (time point when getting out of bed). Sleep duration (h) is the sum of all epochs scored as sleep during the total time spent in bed. Daily sleep data were averaged during each 2-week period in each participant.

© 2022 Tasali E. et al. *JAMA Internal Medicine.*

eTable 2. Effect of Treatment on Actigraphy-Based Outcomes

	Control group (n=40)			Extension group (n=40)				
**Variable**	**Baseline habitual sleep**	**Habitual sleep**	**Change from baseline**	**Baseline habitual sleep**	**Sleep extension**	**Change from baseline**	**Differences in changes (95% CI)**	***P* value**
Time in bed, min	412.34 (403.10 to 421.58)	414.83 (405.58 to 424.07)	2.49 (-7.08 to 12.05)	404.21 (395.08 to 413.34)	490.17 (480.85 to 499.48)	85.96 (76.33 to 95.58)	83.47 (69.90 to 97.04)	<0.001
Sleep duration, min	363.24 (354.17 to 372.30)	364.05 (354.98 to 373.12)	0.81 (-8.51 to 10.13)	355.39 (346.35 to 364.4)	426.64 (417.50 to 435.77)	71.25 (61.78 to 80.71)	70.43 (57.15 to 83.72)	<0.001
Sleep efficiency, %	88.19 (87.02 to 89.35)	88.03 (86.87 to 89.20)	-0.15 (-1.24 to 0.93)	87.74 (86.58 to 88.89)	87.02 (85.85 to 88.19)	-0.72 (-1.82 to 0.39)	-0.56 (-2.11 to 0.99)	0.48
Sleep latency, min	8.08 (6.10 to 10.07)	8.26 (6.27 to 10.24)	0.17 (-1.88 to 2.23)	8.03 (6.05 to 10.01)	12.05 (10.05 to 14.05)	4.02 (1.93 to 6.10)	3.84 (0.92 to 6.77)	0.01
Wake after sleep onset, min	28.62 (25.03 to 32.21)	28.14 (24.56 to 31.73)	-0.47 (-3.00 to 2.05)	29.64 (26.08 to 33.20)	38.41 (34.83 to 41.99)	8.77 (6.20 to 11.35)	9.25 (5.64 to 12.85)	<0.001
Final morning awakening, min	11.80 (9.89 to 13.71)	13.68 (11.77 to 15.59)	1.88 (-0.51 to 4.28)	11.41 (9.50 to 13.31)	12.98 (11.05 to 14.91)	1.57 (-0.85 to 4.00)	-0.31 (-3.72 to 3.10)	0.86
Nap duration, min	5.23 (1.98 to 8.49)	6.29 (3.03 to 9.54)	1.05 (-3.42 to 5.53)	11.04 (7.79 to 14.30)	0.71 (0 to 4.01)	-10.33 (-14.83 to -5.82)	-11.38 (-17.73 to -5.03)	<0.001
Daytime activity, count/min	252.7 (229.6 to 275.8)	259.2 (236.2 to 282.3)	6.5 (-1.9 to 14.9)	262.8 (240.0 to 285.6)	273.1 (250.3 to 296.0)	10.3 (1.7 to 18.9)	3.8 (-8.3 to 15.8)	0.54

Data were from model-derived estimates and reported as mean (95% CI). *P* values for the differences in changes (extension group minus control group) were from the test of the treatment group by period interaction using a linear mixed-effects model approach. All available data were used in the analyses. Bedtime (clock time) is the time point when first lying down in bed with the intent to go to sleep. Wake-up time (clock time) is the time point when getting out

© 2022 Tasali E. et al. *JAMA Internal Medicine.*

of bed at the end of time in bed. Time in bed (minutes) is the total time spent in bed between bedtime and wake-up time. Sleep duration (minutes) is the sum of all epochs scored as sleep during the total time spent in bed. Sleep efficiency (percentage) is the total sleep duration divided by the total time spent in bed multiplied by 100. Sleep latency (minutes) is the time before sleep onset after the bedtime i.e., first lying down in bed. Wake after sleep onset (minutes) is the total number of epochs scored as wake after sleep onset and before final morning awakening. Final morning awakening (minutes) is the wake period at the end of sleep period before getting out of bed in the morning. The nap duration (minutes) is calculated by actigraphy for days that included any naps (i.e., 6% of all available 24-hour actiwatch data) and considered as zero for participants who did not have any naps at all. Daily sleep data were averaged during each 2-week period in each participant.

© 2022 Tasali E. et al. *JAMA Internal Medicine.*

eTable 3. Baseline Characteristics of Participants With Complete vs. Incomplete Data

	Complete data (n=67)	Incomplete data (n=13)	p value
Age, mean (SD), years	29.5 (5.1)	31.2 (4.9)	0.26
Sex, No. (%)			0.84
Male	34 (50.7)	7 (53.8)	
Female	33 (49.3)	6 (46.2)	
Body mass index, mean (SD), kg/m^2	28.1 (1.5)	28.0 (1.0)	0.90
Race/Ethnicity, No. (%)			0.83
White	34 (50.7)	7 (53.8)	
Black or African American	17 (25.4)	3 (23.1)	
Hispanic	6 (9.0)	0 (0)	
Asian	3 (4.5)	1 (7.7)	
More than one Race	7 (10.4)	2 (15.4)	
Employment status, No. (%)			0.38
Full-time employed	43 (64.2)	6 (46.2)	
Part-time employed	4 (6.0)	2 (15.4)	
Student	11 (16.4)	2 (15.4)	
Working from home	5 (7.5)	2 (15.4)	
Unemployed	4 (6.0)	1 (7.7)	
MEQ score, mean (SD) [a]	50.9 (7.8)	53.1 (5.8)	0.25
CES-D score, mean (SD) [b]	6.1 (5.4)	3.3 (4.1)	0.05
TFEQ score, mean (SD) [c]			
Cognitive restrain	8.7 (3.2)	9.2 (3.3)	0.64
Disinhibition	5.4 (3.3)	4.5 (2.4)	0.26
Hunger	4.1 (2.8)	3.9 (2.3)	0.80
Regular exercise, No. (%) [d]	32 (47.8)	6 (46.2)	0.92
Habitual sleep duration, mean (SD), hours [e]	6.0 (0.6)	5.9 (0.5)	0.58

Complete data indicates no missing data for all reported outcomes. Abbreviations: MEQ: Morningness-Eveningness Questionnaire; CES-D: Center for Epidemiologic Studies Depression scale; TFEQ: Three Factor Eating Questionnaire. [a] The score on the 19-item measure ranges from 16 to 86: scores of 16 to 41 indicate evening type (definitely or moderately evening type), scores of 59 to 86 indicate morning type (definitely or moderately evening type) and scores of 42 to 58 indicate intermediate or neither types. [b] The score on the 20-item measure ranges from 0 to 60, scores of 16 and above indicate a higher frequency of depressive symptoms. [c] The score on the 51-item measure ranges from 0 to 20 for Factor I, 0 to 16 for Factor II, and 0 to 15 for Factor III, with higher

© 2022 Tasali E. et al. *JAMA Internal Medicine.*

scores indicating greater levels of restraint, disinhibition and perceived hunger, respectively. [d] Regular exercise was self-reported and defined as engaging in exercise more than twice in an average week and accumulating at least 90 min of moderate or 40minutes of vigorous exercise. [e] Habitual sleep duration was from 1-week wrist actigraphy monitoring during screening.

© 2022 Tasali E. et al. *JAMA Internal Medicine.*

eTable 4. Self-Reported Outcomes by Visual Analog Scales

Variable	Control group (n=40)	Extension group (n=40)	Differences between groups (95% CI)	P value
**Overall, during the past 2 weeks, you:**				
Got sufficient sleep as you needed?	68.3 (21.7)	85.7 (17.4)	17.4 (8.5 to 26.2)	<0.001
Got enough sleep to function at your best during workdays?	73.7 (17.7)	84.6 (18.0)	10.9 (2.8 to 18.9)	0.009
Got enough sleep to function at your best during non-workdays?	82.0 (16.1)	86.1 (17.2)	4.2 (-3.4 to 11.7)	0.28
Had good night's sleep on almost every night?	62.5 (23.4)	78.5 (14.7)	16.0 (7.2 to 24.8)	<0.001
Had more energy during the day?	57.8 (17.9)	79.1 (18.9)	21.4 (13.1 to 29.7)	<0.001
Felt more alert during the day?	57.4 (17.7)	79.7 (19.0)	22.3 (14.1 to 30.6)	<0.001
Were in a better mood or felt happier?	55.8 (18.4)	72.2 (19.9)	16.4 (7.7 to 25.0)	<0.001
Could not complete household chores as much as you wanted?	27.3 (27.2)	30.4 (27.5)	3.1 (-9.2 to 15.5)	0.62
Could not do activities outside home as much as you wanted?	30.5 (23.9)	38.9 (29.0)	8.4 (-3.6 to 20.5)	0.17
Could not watch TV or use internet as much as you wanted?	18.6 (18.6)	42.4 (28.9)	23.8 (12.8 to 34.9)	<0.001
Could not do work related to your job as much as you wanted?	22.2 (19.6)	31.4 (33.8)	9.2 (-3.4 to 21.9)	0.15

Data were reported as mean (SD) unless otherwise specified. At the end of 4-week study, participants were asked questions using visual analog scales about their experience during the preceding 2-week i.e., intervention period. The visual analog scales (score 0 to 100) included a straight line extending from left end of the scale (i.e., score 0, “Strongly disagree”) to the right end of the scale (i.e., score 100, “Strongly agree”). Participants completed the visual analog scales in REDCap by marking any point on the continuous line that corresponds to their subjective agreement to the questions. P values for the differences between groups (extension group - control group) are from two sample t-tests assuming unequal variances (Satterthwaite’s approximation).

© 2022 Tasali E. et al. *JAMA Internal Medicine.*
