# Supplementary material for: Effect of Sleep Extension on Objectively Assessed Energy Intake Among Adults With Overweight in Real-life Settings: A Randomized Clinical Trial
Source: JAMA Intern Med. 2022 Feb 7;182(4):365–74. doi: 10.1001/jamainternmed.2021.8098 (PMC8822469; doi:10.1001/jamainternmed.2021.8098)
Supplement: Supplement 3. — Data Sharing Statement [file jamainternmed-e218098-s003.pdf]

Data Sharing Statement

Tasali. Effect of Sleep Extension on Objectively Assessed Energy Intake Among Adults With Overweight in Real-Life Settings. *JAMA Intern Med*. Published February 07, 2022. doi:10.1001/jamainternmed.2021.8098

Data
Data available: No

Additional Information
Explanation for why data not available: Data will be shared in clinical trial.gov after publication
